# Myrrh Oil *in Vitro* Inhibitory Growth on Bovine and Equine Piroplasm Parasites and *Babesia microti* of Mice

**DOI:** 10.3390/pathogens9030173

**Published:** 2020-02-29

**Authors:** Mahmoud AbouLaila, Shimaa Abd El-Salam El-Sayed, Mosaab A. Omar, Mohammad Saleh Al-Aboody, Amer R. Abdel Aziz, Mohamed M. Abdel-Daim, Mohamed Abdo Rizk, Ikuo Igarashi

**Affiliations:** 1 National Research Center for Protozoan Diseases, Obihiro University of Agriculture and Veterinary Medicine, Inada-Cho, Obihiro, Hokkaido 080-8555, Japan; hethet2004@yahoo.com (M.A.); abdo_dr_shimaa@yahoo.com (S.A.E.-S.E.-S.); igracipmi@obihiro.ac.jp (I.I.); 2 Department of Parasitology, Faculty of Veterinary Medicine, Damanhour University, Damanhour 22511, ElBehera, Egypt; 3 Department of Biochemistry and Chemistry of Nutrition, Faculty of Veterinary Medicine, Mansoura University, Mansoura 35516, Egypt; 4 Department of Veterinary Medicine, College of Agriculture and Veterinary Medicine, Qassim University, Buraydah, 51452 Qassim, Saudi Arabia; mos.mohamed@qu.edu.sa; 5 Department of Parasitology, Faculty of Veterinary Medicine, South Valley University, Luxor 83523, Qena, Egypt; 6 Department of Biology, College of Science in Zulfi, Majmaah University, Majmaah 11952, Saudi Arabia; m.alaboudi@mu.edu.sa; 7 Department of Parasitology, Faculty of Veterinary Medicine, Sohag University, Sohag 82524, Egypt; amerragheb36@yahoo.com; 8 Department of Internal Medicine and Infectious Diseases, Faculty of Veterinary Medicine, Mansoura University, Mansoura 35516, Egypt; 9 Department of Zoology, College of Science, King Saud University, P.O. Box 2455, Riyadh 11451, Saudi Arabia; 10 Pharmacology Department, Faculty of Veterinary Medicine, Suez Canal University, Ismailia 41522, Egypt

**Keywords:** Myrrh oil, *Babesia*, *T. equi*, *in vitro*, *in vivo*

## Abstract

The present experimental study was conducted for the assessment of the efficacy of in vitro inhibition of myrrh oil on the propagation of *Babesia* *bovis, B. divergens, B. bigemina, Theileria equi,* and *B. caballi* and in vivo efficacy on *B. microti* in mice through fluorescence assay based on SYBR green I. The culture of *B. divergens* *B. bovis* and was used to evaluate the in vitro possible interaction between myrrh oil and other commercial compound, such as pyronaridine tetraphosphate (PYR), diminazene aceturate (DA), or luteolin. Nested-polymerase chain reaction protocol using primers of the small-subunit rRNA of *B. microti* was employed to detect any remnants of DNA for studied parasitic species either in blood or tissues. Results elucidated that; Myrrh oil significantly inhibit the growth at 1% of parasitic blood level for all bovine and equine piroplasm under the study. Parasitic regrowth was inhibited subsequently by viability test at 2 µg/mL for *B. bigemina and* *B. bovis*, and there was a significant improvement in the in vitro growth inhibition by myrrh oil when combined with DA, PYR, and luteolin. At the same time; mice treated with a combination of myrrh oil/DA showed a higher inhibition in emitted fluorescence signals than the group that challenged with 25 mg/kg of diminazene aceturate at 10 and 12 days post-infection. In conclusion, this study has recommended the myrrh oil to treat animal piroplasmosis, especially in combination with low doses of DA.

## 1. Introduction

*Babesia* and *Theileria* are tick-borne protozoal parasites which infect the erythrocytes of domesticated animals, and it is incriminated in producing significantly high economic losses in the livestock industry and animal trade all over the world [1]. The clinical picture of the disease is typically associated with hyperthermia, malaise, yellowish discoloration of all mucous membranes, especially conjunctiva, hemoglobin in the urine, and occasionally, the infected animal died [2]. *B. divergens Babesia bovis*, and *Babesia bigemina* were considered the main causative agents of babesiosis in cattle, that provoking a huge loss on the animal health and productivity [2]. Besides, *B. divergens* has public health significance and serious zoonotic importance in Europe [3]. On the other hand; *Babesia caballi* and *Theileria equi* considered the main causative agents in piroplasmosis of equines [4], but Unfortunately, bovine and equine *Babesia* infection have no suitable laboratory experimental animals to induce the in vivo studies for this parasites, and there are alternative that was conducted and established by scientists which are the mouse model infected with *B. microti,* which is rodent *Babesia*, and it was found that it is suitable for antibabesial drug evaluation against different *Babesia spp.,* that infecting domestic and farm animals, since the inhibitory effect of the newly developed drug must be firstly evaluated in laboratory animals to determine the possible adverse effect of these hits before it is administered to animals under field condition [5]. Currently, the commercially available anti-piroplasm drugs have shown a toxic effect to the infected animal as imidocarb dipropionate as nausea, salivation, drowsiness, and recumbency, also other drugs as diminazene aceturate (DA) developed some degree of resistance from the treated parasite [1]. Accordingly, the discovery of alternatives that have more significant efficacy and safe anti-piroplasm agents is mandatory. In this attitude, using herbal therapies or compounds that extracted from the natural products might be an alternative strategy, such as the herb myrrh (*Commiphora molmol*), which has shown several clinical benefits, that is mainly attributed to its oil [6]. Myrrh oil has anti-inflammatory [7], analgesic [8], antischistosomal [9], fasciolicidal [10], antimoniezia [11], molluscicidal [12], acaricidal [13], mosquitocidal [14], antigiardial [15], anticoccidial [16] activities. However, the anti-piroplasm effect of myrrh oil was not yet experimentally conducted. Hence, this trial evaluated the potent inhibitory effect of myrrh oil as an anti-piroplasm candidate to inhibit the growth of bovine and equine piroplasmosis, and for Babesia microti in mice as an experimental model. 

## 2. Results

### 2.1. In vitro Growth Inhibition Assay 

**Figure 1:**
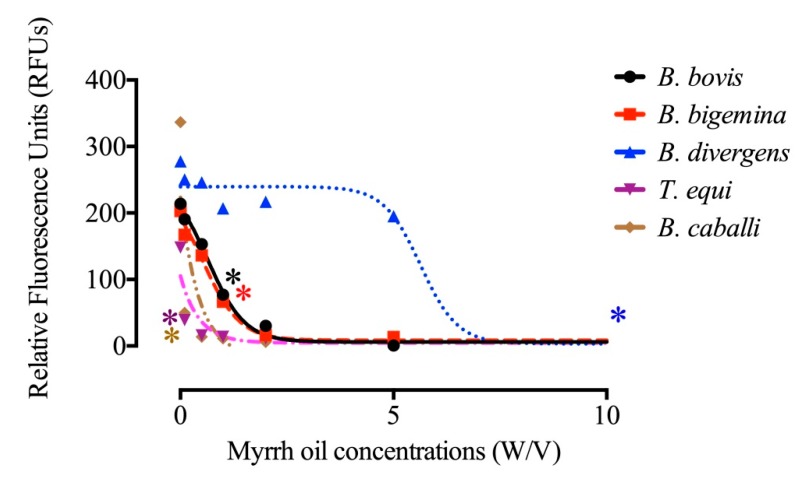
Correlation between the log concentrations and relative fluorescence units (RFUs) of myrrh oil (µg/mL) on *Babesia bigemina, Babesia bovis, B. divergens, B. caballi*, and *T. equi* on the fourth day p.i. Every value is considered the mean of triplicate wells after subtraction of the background fluorescence for non-parasitized RBCs. Asterisks indicate a significant difference (ANOVA; * *P* < 0.05) between the myrrh oil-treated and the control cultures.

The IC_50_s of myrrh oil revealed that the in vitro inhibitory effect of myrrh oil has a highly significant efficacy and susceptibility on equine piroplasm parasites than those observed in bovine Babesia parasites (Table 1), and the myrrh oil IC_50_ values to *T. equi* was similar to DA IC_50_ values (Table 1). 1 μg/mL myrrh oil significantly inhibits the in vitro growth of *Babesia bigemina, Babesia bovis, B. caballi, and T. equi* (*P* < 0.05) (Figure 1), but on the contrary, significantly inhibited *B. divergens* in vitro growth (*P* < 0.05) at 10 μg/mL (Figure 1). For regrowth assay; it was noted that *Babesia divergens, T. equi and Babesia caballi* were inhibited by viability test with 10 µg/mL (Table 2), and treatment of *Babesia bovis* and *Babesia bigemina* on the culture at 2 µg/mL myrrh oil prevented parasite regrowth (Table 2). 

**Figure 2:**
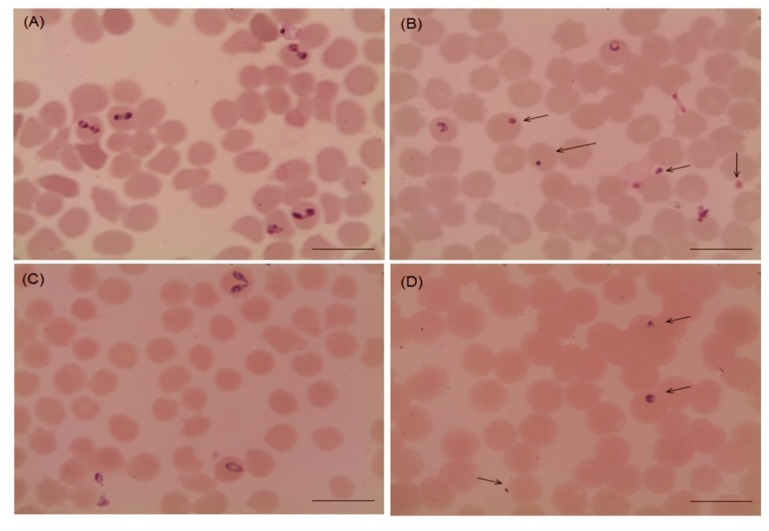
Microscopy of *Babesia bovis* and *Babesia bigemina* handled with 10 µg/mL myrrh oil in cultures. (**A**) *Babesia bovis* control group, (**B**) myrrh oil-treated cultures group, (**C**) *Babesia bigemina* control, and (**D**) myrrh oil-treated cultures. The drug-treated cultures showed higher numbers of degenerated parasites indicated by arrows than the control cultures. Micrographs were taken on day 4 of treatment. *Scale bars* 10 μm.

**Figure 3:**
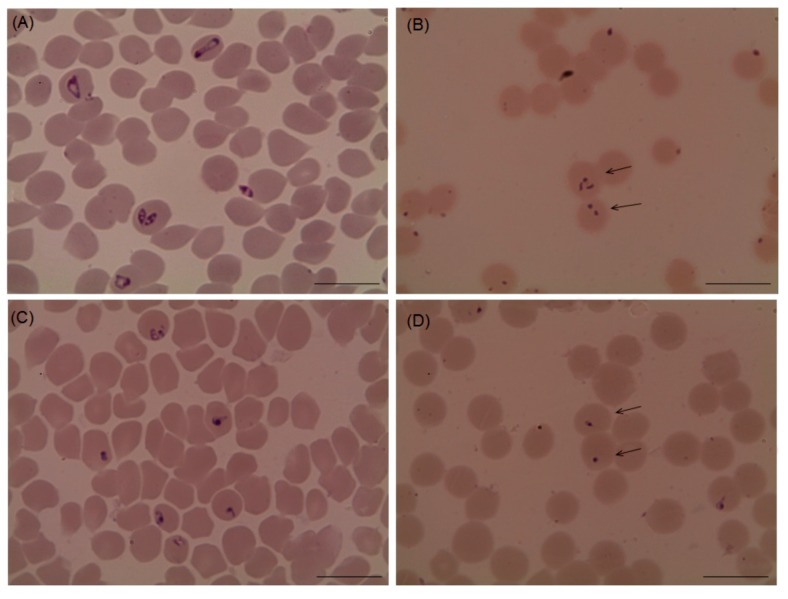
microscopic examination of *Babesia caballi* and *Theileria equi* treated with 10 µg/mL myrrh oil cultures. (**A**) *Babesia caballi* control, (**B**) myrrh oil-treated cultures, (**C**) *Theileria equi* control, and (**D**) myrrh oil-treated cultures. The drug-treated cultures showed higher numbers of degenerated parasites indicated by arrows than the control cultures. Micrographs were taken on day 4 of treatment. *Scale bars* 10 μm.

Several morphological changes in the parasites were observed after the treatment with myrrh oil in comparison with that recorded from the control cultures (Figure 2 and Figure 3), in which, parasites appeared degenerated from cultures of *Babesia bovis* (Figure 2B), *Babesia bigemina* (Figure 2D), *Babesia caballi* (Figure 3B), and *Thieleria equi* (Figure 3D) by light microscope. The pattern of parasitic growth in cultures using myrrh oil 250 µg/mL pretreated erythrocytes was similar to cultures used non-pretreated erythrocytes. Furthermore, erythrocyte morphology in pretreated cultures was similar to those the non-treated erythrocytes by light microscope (observations not listed). 

### 2.2. Drug Combination Test 

The significant in vitro inhibitory effect of myrrh oil combined with luteolin, DA, and pyronaridine tetraphosphate (PYR) on *B. divergens* and *B. bovis* was tested in this study, and results showed that, the inhibition rate of *B. bovis* was significantly high when treated with myrrh oil combined with DA, PYR, and luteolin at 0.50 IC_50_ myrrh oil and 0.12 IC_50_ DA, 0.12 IC_50_ myrrh oil and 0.50 IC_50_ PYR, and 0.25 IC_50_ myrrh oil and 0.50 IC_50_ luteolin (Table 3, Table 4, and Table 5). These findings confirmed the potential anti-*B. bovis* efficacy of myrrh oil, especially when administrated simultaneously in lower doses with other common antipiroplasma drugs, but on the contrary, combination of myrrh oil with other therapies did not enhance the in vitro inhibitory effect on *B. divergens*, and it was found that it is of no significant value (Table 3, Table 4, and Table 5). 

### 2.3. Chemotherapeutic Efficacy of Myrrh Oil on B. Microti Infection

**Figure 4:**
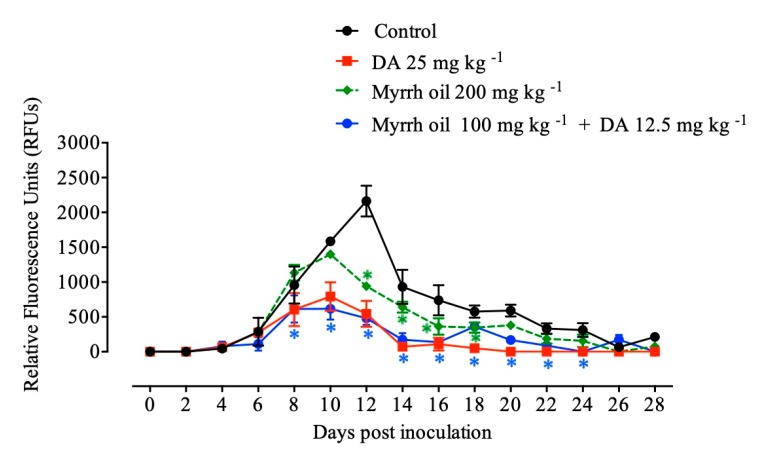
Inhibition of growth by myrrh oil, diminazene aceturate (DA), and both drugs combined on *Babesia microti*. Every value is considered the mean ± standard deviation of five mice per experimental group. Asterisks indicate significant differences (ANOVA; * *P* < 0.05) between the myrrh oil–treated and control groups.

The efficacy of in vivo inhibition of myrrh oil was detected against *Babesia microti* in a mouse model and explored that; myrrh oil alone exhibited inhibition (*P* < 0.05) in the emitted fluorescence signals from 12th–18th days p.i. in comparison with the positive control group. Peak fluorescence values were detected in the treated groups as 793.50, 1399, and 616.50 with 25 mg kg^-1^ diminazene aceturate, 200 mg kg^-1^ myrrh oil, also 100 mg kg^-1^ myrrh oil in combination with 12.5 mg kg^−1^ dimenazine aceturate at 10th days post infection, respectively, in contrary, positive control group, the average of maximum fluorescence values was 2161.11 at 12 days p.i. The intraperitoneal injections with 200 mg kg^−1^ for five successive days achieved 56.57% inhibition compared with 74.83% inhibition at 25 mg kg^−1^ DA at 12th day post infection. Of note, the inhibition in the fluorescence values were higher in mice manipulated with myrrh oil/diminazene aceturate combination than those obtained from mice treated with 25 mg kg^−1^ DA alone at 10 and 12 days p.i. Intraperitoneal injections of 100 mg kg^-1^ myrrh oil in combination with subcutaneous dose 12.5 mg kg^−1^ DA caused 61.08% and 77.80% inhibitory effect of growth at 10th and 12th days p.i., respectively compared with 49.91% and 74.83% inhibitions at 25 mg kg^−1^ DA at days 10 and 12 p.i., respectively (Figure 4). 

**Figure 5:**
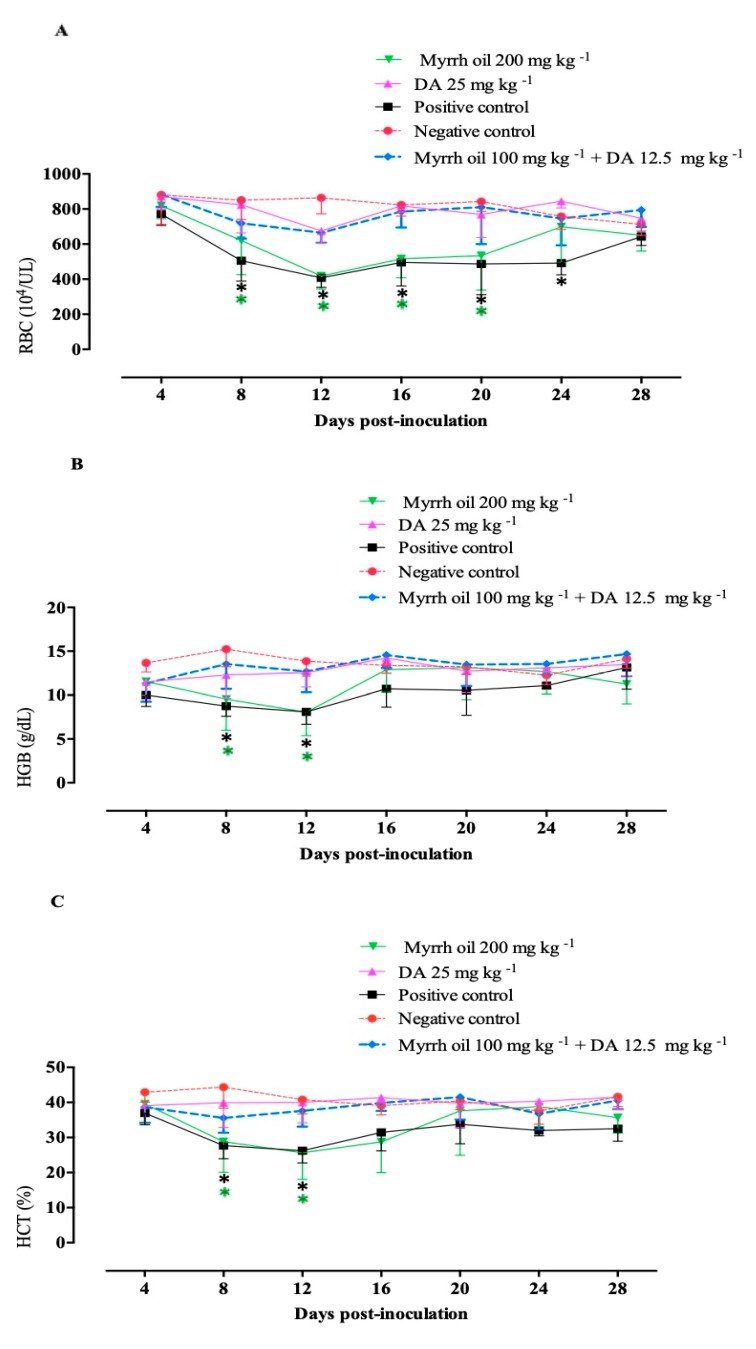
Hematological variables in *B. microti*- infected mice subjected to myrrh oil. (**A**) RBCs. (**B**) Hemoglobin (HGB). (**C**) Hematocrit (HCT). Each value is the mean ± standard deviation of five mice per experimental group. Asterisks indicate a significant difference (ANOVA; * *P* < 0.05) between the treated or infected mice and the uninfected mice.

Mice that treated with a combination of myrrh oil with a low dose of Diminazene aceturate normalized the assessed hematological variables in similar to groups treated with 25 mg kg^−1^ DA (Figure 5). Such findings revealed the promising antibabesial efficacy of myrrh oil/DA combination therapy. 

**Figure 6:**
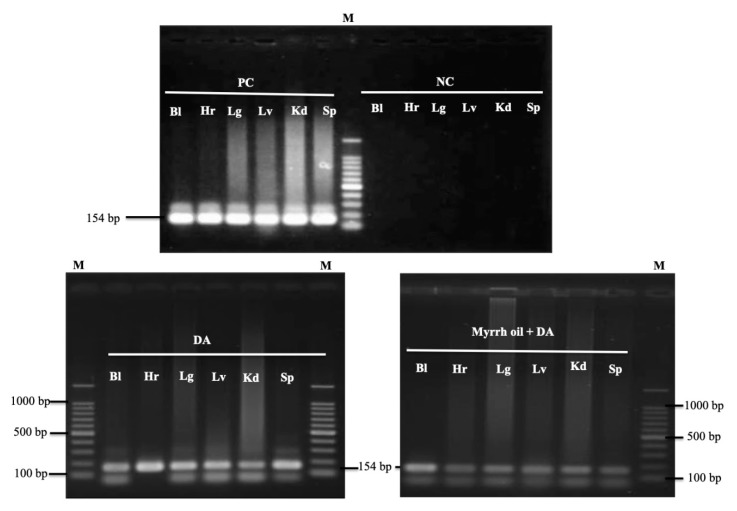
PCR of the *ss-rRNA* gene in blood and different organs of *B. microti–*infected mice treated with ethanol (positive control), 25 mg kg^−1^ diminazene aceturate (DA), and DA (12.5 mg kg^−1^) combined with myrrh oil (100 mg kg^−1^). PC, positive control; NC, negative control; Bl, blood; Hr, heart; Lg, lung; Lv, liver; Kd, kidney; Sp, spleen, M indicates a 100 bp DNA ladder.

On day 28 p.i. a nested PCR protocol of *B. microti* using small subunit rRNA (ss-rRNA) gene to detect any remnant of the parasites in the blood samples and tissues of experimental mice. Unfortunately, positive results obtained—indicating that the parasite gene was found in the blood and all examined tissues from treated groups of mice with Diminazene aceturate alone or combined with myrrh oil (Figure 6). 

## 3. Discussion

In the current study, the inhibitory effect of myrrh oil against the growth of *B. bovis, B. bigemina, B. divergens, T. equi* and *B. caballi* in vitro and against *B. microti* in vivo was evaluated. IC_50_ values of myrrh oil for *Babesia* species and *T. equi* were very low compared with the IC_50_ value for *P. falciparum* (100 µg/mL) [17]. IC_50_ values of myrrh oil for *Babesia* species and *T. equi* were nearly similar to epoxomicin [19]. Interestingly, IC_50_ values of myrrh oil for *Babesia* species and *T. equi* were lower than those of quinuronium sulfate [20], imidocarb dipropionate [21], heparin [22], curdlan sulfate [23], triclosan [24], (-)-epigallocatechin-3-gallate [25], nerolidol [26], ciprofloxacin [27], fusidic acid [28], miltefosine [29], allicin [30], N-acetyl-L-cysteine [31], and thymoquinone [32]. Additionally, the IC_50_ value of myrrh oil for *B. divergens* was lower than those of fluoroquinolones, including enrofloxacin, enoxacin, trovafloxacin, norfloxacin, and ofloxacin [33]. IC_50_ values of myrrh oil for piroplasm parasites were very low compared with the drug’s IC_50s_ for mammalian cells, such as MCF-7 (19.8 µg/mL), HepG2 (39.2 µg/mL), Hela (34.3 µg/mL), HS-1 (22.7 µg/mL), and A459 (41.4 µg/mL) [18], highlighting the high selectivity index of myrrh oil. Furthermore, in the current study, the very high concentration of myrrh oil did not affect the bovine or equine RBCs. Such findings confirm the non-toxic effect of myrrh oil. The issue that indicates the safety of myrrh oil on the in vitro studies level and recommends the further use of this promising anti-piroplasm candidate in the in vivo studies.

Some herbs were evaluated against babesiosis and theileriosis in vitro, *such as* green tea, black tea, hibiscus, cinnamon, and peppermint [34], and flavonoids isolated from the flowers of *Pulsatilla flavescens* [35].

In our study, in combination with DA, luteolin and PYR, the in vitro inhibitory effect of myrrh oil was increased against *B. bovis* growth. These findings are analogous to allicin/Da [30] and TQ/DA in vitro inhibitory actions on *B. bovis* [32]. The effect of myrrh oil on in vitro inhibition if used together with DA, PYR or luteolin, *B. divergens* was not improved. *In vitro B. divergens* suppression of development, on the other hand, in blend with DA, luteolin, and PYR were increased by treatment with a fluoroquinolone (enoxacin, enrofloxacin, and trovafloxacin) [33]. Such non-improvement in the inhibitory effects of myrrh oil on *B. divergens*, when used in combination with the selected antibabesial drugs, might be attributed to the fact that parasite species, strain, and size affect the in vitro inhibitory activity of the drug combination on the *Babesia* growth [30,36,37,38].

The apparent effect on *Babesia* and *Theileria*, as an in vitro inhibitor, has led us to test its in vivo inhibitory effect on *B. microti*. Results showed that lower doses of the mixture of myrrh oil/DA are in general more active than a high DA dose alone.

Inhibition of *B. Microti* development caused by a mixture of 100 mg kg^−1^ myrrh oil and 12.5 mg kg^−1^ DA is higher than 70 percent inhibition rate for clindamycin/ quinine mixture [34], 56.35 and 53.25 percent inhibition rate for the 85 mg kg^−1^ PYR joined with 10 mg kg^-1^ DA [35], 67 percent hang-up rate for the 50 mg kg^−1^ enoxacin and 10 mg kg^−1^ DA, and 62.5 percent inhibition rates for 50 mg kg^–1^ oral dose of TQ and 10 mg kg ^-1^ subcutaneous dose of DA [32].

The nested PCR assay was performed to assess the capacity of the administered combination therapy to further remove parasite DNA from the organs of the animal. PCR magnification observed by the *B. microti* ss-rRNA gene in the blood and organs of mice cured with myrrh oil/DA combination. Likewise, the PYR/DA combination did not completely eliminate the infection from different organs of *B. micro*-infected animals on the thirtieth day post-infection [35].

Conversely, combination therapy consists of enoxacin/DA removed *B. microti* infection from the lungs of treated mice, but not from other organs (spleen, heart, and kidney) on day 20 p.i. [33]. This nucleic acid residue of the parasite that has been found in the blood and organs of the treated mice in our sample highlights the failure of each DA alone and DA/myrrh oil combination to totally remove parasite DNA from the animal body on 28th-day p.i., may subsequently be reinfected.

Future research is, therefore, desired to determine the in vivo inhibiting results of a safe anti-piroplasm agent, myrrh oil when used in combination with DA, low doses of imidocarb dipropionate, or recently developed anti-piroplasm drugs, such as clofazimine [36], PYR, or luteolin [16,37] for longer durations of infection.

Myrrh oil contains many active compounds, such as sesquiterpenoids, including furanodiene, 1(l0)Z,4Z-furanodiene-6-one(1), 2-methoxyfuranodiene, 2-acetoxyfuranodiene, 4,5-dihydrofuranodtene-6-one, 2-methoxyfuranoguaia-9-ene-8-one, isofuranogermacrene (1), lindestrene (2), furanoeudesma-l,3-diene (3), furanodiene (4), and nerolidol [18,39,40]. The antibabesial effect of myrrh oil may be attributed to one or more constituents, such as terpen nerolidol that was reported as a potent antibabesial agent [26,41].

Although the present study has shown an improvement in the in vitro inhibitory effect of myrrh oil when used in combination with DA, PYR, and luteolin on *B. bovis* growth, synergetic or antagonistic relationships between these combination therapies have not yet been assessed against *B. microti* growth on the rats. Additional experiments are, therefore, required to decide the possible inhibitory results of these combinations on *B. microti* development in the mouse model. Extra, future research is necessary to elucidate the mechanism for improving the inhibitory effect of myrrh oil when used in association with DA, PYR, or luteolin. This study evaluated the inhibitory effect of myrrh oil as whole oil, and neglected the purification of the oil compounds. Subsequently, future studies are required to purify the components of myrrh oil and test their inhibitory effects against the growth of *Babesia* parasites separately. Although the present study evaluated the in vivo inhibitory effects of myrrh oil when used in combination with DA, the inhibitory effect of DA when administrated as monotherapy at a dose rate of 12.5 mg kg^−1^ was not evaluated. Thus, further studies are warranted to evaluate the inhibitory effect if DA (12.5 mg kg^−1^) alone to rule out the possibility that DA at this dose may cause similar efficacy as the combination therapy. 

## 4. Materials and Methods 

### 4.1. Chemicals

SYBR Green I (SGI) nucleic acid stain (Lonza, Rockland, USA; 10,000x) was processed at −20 °C and warm-up prior to using. A lyse buffer involving Tris (130 mM; pH 7.5), EDTA (10 mM), saponin (0.016per cent; W/V), and TritonX-100 (1.6%; V/V) was ready previously and stockpiled at 4 °C. Myrrh oil has been obtained from Sigma-Aldrich (Saint Louis, USA). 100 mg/mL stock solutions in Ethanol (Eth) were ready and stockpiled at −30 °C before use. DA (Ganaseg, Ciba-Geigy Japan Ltd., Tokyo, Japan) is a widely put to use antibabesial drug used as a confident regulator drug. A functioning store solution of 10 mM of DA or luteolin, and pyronaridine tetraphosphate (PYR) (Sigma-Aldrich, Japan) has been dissolved in double-distilled water (DDW) and stockpiled at −30 °C until needed for usage. 

### 4.2. In Vitro Growth Inhibition Assay and Viability Test 

Myrrh oil was tested for its chemotherapeutic activity against *B. bovis* (Texas strain) [16], *B. bigemina* (Argentina strain) [23], *B. caballi* [26], and *T. equi* (United States Department of Agriculture) [4]. Parasites have been cultured in bovine or equine red blood cells using a continuous microaerophilic stationary phase culture system [16,23]. The inhibitory results of myrrh oil on the growth of *Babesia/Theileria* was checked by fluorescence assay using SGI stain [23,42]. Double 96-well plates (Nunc, Roskilde, Denmark) were used for cultivating bovine *Babesia* and equine *Babesia/Theileria* pRBCs using medium alone or with defined concentrations—0.1, 0.5, 1.0, 2, 5, or 10 µg/mL for myrrh oil. The concentrations used have been based on a preliminary study. Positive control cultures earned 0.1, 0.5, 1.0, 2, 5, and 10 µg/mL of DA. Of negative experimental controller, non-drug cultures and cultures containing only Eth (0.0025 per cent of myrrh oil) and DDW (0.02 per cent for DA) were prepared. Bovine *Babesia* and equine *Babesia/Theileria* parasites pRBCs were cultured at 1% parasitemia in 96-well plates using 2.5 percent HCT for *B. bovis* and *B. bigemina* and 5% HCT for further *Babesia* and *Theileria* parasites. The pRBCs were cultured for four days in triplicate wells for each concentration of the drug. On the fourth day of cultivation, the IC_50_ values were calculated by adding lyse buffer containing 2× SGI to every drug dilution on the first 96-well plate. While, for the second plate, 1.5 µL (for parasites cultivated with 5 per cent HCT) and 0.75 µL (for parasites cultivated with 2.5 per cent HCT) of each of the control and drug-treated infected RBCs were mixed with 3.5 µL of parasite-free RBCs and 1.75 µL for parasites cultivated with 5 per cent and 2.5 per cent HCT, respectively on the fourth day of cultivation [22]. Afterwards, bovine and equine packed red blood cells were overhung in a new medium with no drug. The plates were then incubated at 37 °C for the next 4 days without the medium being replaced. The first or second plate then kept warm for 6 h in a dark dwelling at 25 °C, and fluorescence appraisals were calculated via a fluorescence plate reader (Fluoroskan Ascent, Thermo Electron Informatics, Philadelphia, PA, USA) at 485 nm and 518 nm wavelengths, respectively. Gain values have been fixed at 100. That research has been threefold replicated. 

### 4.3. Myrrh Oil in Combination with Other Antibabesial Drugs in Vitro

The combination of myrrh oil therapies with commonly used anti-piroplasm drugs, DA, and the recently developed anti-piroplasm drugs, luteolin or PYR [16] was examined in vitro cultures of *B. bovis* and *B. divergens* (the parasites that showed the highest IC_50_ for myrrh oil). Myrrh oil/DA (M1, M2, M3, M4, M5, and M6), luteolin or PYR mixtures (M1, M2, M3, M4, M5, M6, M7, M8, and M9) were ready as mentioned before [16] with certain reforms. Combinations were founded on the measured IC_50_ values extracted from the in vitro fluorescence assay. Drug-free cultures have been used for experimental monitoring. Cultures containing only DA, luteolin or PYR IC_50s_ of the parasite were used as positive drug monitors. Three separate trials were conducted, consisting of three-fold drug combination experiments over a 4-day period using 2.5 per cent and 5 per cent HCT for *B. bovis* and *B. divergens,* respectively. The fluorescence values were determined on the fourth day of cultivation, by tallying lytic buffer to every drug combination as mentioned above.

### 4.4. Determination of Morphological Changes and the Toxic Effect of Myrrh Oil on Host Erythrocytes in Vitro

Morphological fluctuations in drug-exposed *Babesia* and *T. equi* parasites were seen using a microscope [23,27]. The experiment was conducted at a parasitemia of 1% for all parasites. The growth inhibition assay was completed in 96-well plates at 10% HCT (20 µL of red blood cell inoculum and 200 µL of the correct medium), and the parasites were treated with 10 µg/mL of myrrh oil. The plates were cultivated for four consecutive days, as mentioned above. Variations in the morphology of drug-exposed *Babesia* species have been determined by the use of light microscopy in a Giemsa-stained thin erythrocyte smear coating and contrasted with the controller. 

The poisonousness of myrrh oil to host erythrocytes was assessed by way of mentioned formerly [25]. Bovine and equine erythrocytes were incubated either with either medium alone or medium with a very high concentration of myrrh oil (250 μg/mL) for 3 h at 37 °C. RBCs were at that point cleaned three turns with drug-free media and driven for 72 hours. Parasite development in pretreated erythrocytes was witnessed and matched to control untreated cells. Experiments were performed in triple wells for each drug concentration for all parasite species and in three isolated trials.

### 4.5. Chemotherapeutic Efficacy of Myrrh Oil on the Growth of B. Microti in Mice

All in vivo experimental protocols in this study were approved by the Animal Care and Use Committee, Obihiro University of Agriculture and Veterinary Medicine (Approval No. 27-65). All experiments were conducted in accordance with the Fundamental Guidelines for Proper Conduct of Animal Experiment and Related Activities in Academic Research Institutions under the jurisdiction of the Ministry of Education, Culture, Sports, Science and Technology, Japan. 

*In vivo* inhibition of myrrh oil test for *B. microti* (Munich strain) [32] was performed twice in BALB/c mice aged eight weeks old (purchased from CLEA Japan, Tokyo, Japan) using the previously described method [38]. Every mouse was housed under strict pathogen-free conditions. Twenty-five female BALB/c mice were equally divided into five groups. All mice were injected intraperitoneally with 1 × 10^7^ *B. microti*-infected RBCs except for mice in the first community, persisted uninfected and used as the negative monitor. Once the infected mice revealed around 1% parasitemia, mice in the investigational groups were given regular injections for five days. Myrrh oil was dissolved in Eth (50% v/v), and DA was dissolved in DDW (12.5%), and then weakened in PBS or DDW prior to injection. Mice in the second group were received intraperitoneal doses of Eth in PBS (0.006 per cent) and were used equally inactive governor. DA has been given in subcutaneous doses to the mice in the third group at a rate of 25 mg kg^−1^. Myrrh oil was administered intraperitoneally either only at a dose rate of 200 mg kg^−1^ or in conjunction with a subcutaneous dose of DA at a dosage level of 100 mg kg^−1^ myrrh oil and 12.5 mg kg^−1^ DA for mice in the fourth and fifth groups, respectively. The inhibitory effects of the drugs administered on the growth of *B. microti* were tracked each 48 h until 28 days after infection or the ending of parasitemia using fluorescence spectrophotometer [38]. After completion of the experiment, all the mice were humanely euthanized using inhalational agent, chloroform as a primary method of euthanasia, and cervical dislocation (physical euthanasia) was performed to all mice.

### 4.6. The Potential of Myrrh Oil in the Recovery from the Anemia Accompanying Babesia

HCT estimates, hemoglobin (HGB) levels, RBC counts, were used to determine the ability of myrrh oil to recover the *B. microti*- infected mice with induced anemia. Ten microliter blood samples were possessed from all mice each 96 h and hematological variables were monitored using Celltac α MEK-6450 automatic hematology analyzer (Nihon Kohden Corporation, Tokyo, Japan). 

### 4.7. PCR Detection of B. Microti in Mice 

Blood and tissue parasite DNA in (heart, lung, liver, kidney and spleen) samples possessed from mice cured with myrrh oil/DA mixture, DA alone, and Eth (affirmative control) were identified using nested PCR tests directing the *B. microti small subunit rRNA* (*ss-rRNA*) gene on day 28 after infection (p.i.). A NucleoSpin tissue kit (Macherey-Nagel, Düren, Germany) and a QIAamp DNA Blood Mini Kit (Qiagen, Tokyo, Japan) were used for tissues and blood DNA extraction. PCR cycling was accomplished, as mentioned earlier by Rizk et al. [33,38].

### 4.8. Statistical Analysis

GraphPad Prism 5th edition from GraphPad Software Inc. (CA, USA) was directed to assess the substantial alterations concerning the groups tested using a one-way ANOVA method. The value of *P* < 0.05 was deemed of analytical importance. Statistically noteworthy variances between the drug-exposed and affirmative-controller clusters have been used as an indicator of the recurrence of the parasite in a viability test [42].

## 5. Conclusions

In conclusion, the potential anti-piroplasm effects of myrrh oil have been demonstrated in vitro and in vivo in this research. Noteworthy, myrrh oil once used in conjunction with a low dose of DA, myrrh oil has a higher inhibitory effect on *B. microti* development following treatment with the standard dose (25 mg kg^−1^) of the widely used anti-piroplasm drug, DA. The consequences of this study propose that myrrh oil may be useful for the therapy of animal piroplasmosis, specifically when used in conjunction with a low dose of DA. The issue that might help to avoid the opposition to DA. 

## Figures and Tables

**Table 1:** Evaluation of IC_50_ values of myrrh oil for bovine Babesia, equine Babesia, and Theileria parasites.

Organism	IC_50_ (µg/mL) ^a^	
	Myrrh Oil	Diminazene Aceturate
*Babesia* *bovis*	1.04 ± 0.13	0.18 ± 0.03
*Babesia* *bigemina*	0.96 ± 0.03	0.09 ± 0.005
*Babesia* *divergens*	7.70 ± 0.41	0.08 ± 0.01
*Theileria equi*	0.33 ± 0.10	0.35 ± 0.01
*Babesia* *caballi*	0.18 ± 0.03	0.01 ± 0.001
*Plasmodium falciparum* ^b^	100	ND
HepG2 cells ^c^	39.2	ND

^a^ IC_50_ values for myrrh oil and diminazene aceturate were recorded and monitored at fourth day depends on inhibitory effect on growth by fluorescence-based evaluation in three different groups. Every concentration of used drugs was manipulated in made in triplicate at each experimental group, and IC_50_ consider the mean and standard deviation of three separate treatments. ^b^ Fujisaki et al. [17]. ^c^ Chen et al. [18]. ND, not determined.

**Table 2:** Myrrh oil evaluation for bovine Babesiosis, equine Babesia, and Theileria parasites by viability test results.

Parasite	Drug Concentrations (μg/mL) ^a^					
	0.1	0.5	1.0	2.0	5.0	10
*B. bovis*	+	+	+	−	−	−
*B. bigemina*	+	+	+	−	−	−
*B. divergens*	+	+	+	+	+	−
*T. equi*	+	+	+	+	+	−
*B. caballi*	+	+	+	+	+	−

^a^ any value calculation was dependent on the fluorescence assay in three different treatment of experiments. Every concentration was done in triplicate in each experiment.

**Table 3:** Growth inhibition effect of myrrh oil combined with diminazene aceturate on *B. divergens* and *B. bovis* parasites.

Group	Fluorescence Values (mean ± SD) ^a^	
	*B. divergens*	*B. bovis*
Control	516.46 ± 42.51	220.44 ± 17.28
DA IC_50_	240.01±21.86	121.96 ± 8.30
M1 (0.75:0.50)^b^	424.33 ± 23.72 *	30.59 ± 12.69 **
M2 (0.75:0.12) ^b^	446.65 ± 24.54 *	67.23 ± 13.26 **
M3 (0.50:0.50) ^b^	478.39 ± 35.17	67.07 ± 7.77 **
M4 (0.50:0.12) ^b^	483.60 ± 38.57	95.56 ± 1.48 **
M5 (0.25:0.50) ^b^	481.66 ± 42.51	106.02 ± 25.12 *
M6 (0.25:0.12) ^b^	499.17 ± 41.87	114.05 ± 5.74 *
M7 (0.12:0.50) ^b^	509.22 ± 33.15	120.75 ± 7.51 *
M8 (0.12:0.12) ^b^	513.33 ± 27.66	124.88 ± 10.39 *
M9 (0.06:0.50) ^b^	516.25 ± 21.08	131.02 ± 9.47 *
M10 (0.06:0.12) ^b^	516.10 ± 31.42	140.11 ± 11.08 *

^a^ Every value detected using fluorescence assay in three different experiments. Every concentration of the tested drug combination was done three times in each experimental group, and the abovementioned value considered the mean and standard deviation (SD) of three separate experimental group after subtraction of the background fluorescence for non-parasitized RBCs and multiplied by 100. * *P* < 0.05 statistically significant differences between the combined-drug-treated group and the control group. **P < 0.05 statistically significant differences between the combined-drug-treated group and the both control group and DA- treated group. DA, diminazene aceturate. ^b^ M1- 10 refers to the combinations of myrrh oil combined with DA based on the calculated IC_50_ values obtained from the in vitro fluorescence assay.

**Table 4:** Growth inhibition effect of myrrh oil combined with pyronaridine tetraphosphate on *B. divergens* and *B. bovis* parasites.

Group	Fluorescence Values (mean ± SD) ^a^	
	*B. divergens*	*B. bovis*
**Control**	530.21 ± 14.83	230.44 ± 35.56
**PYR IC_50_**	283.50 ± 20.11	127.31 ± 14.21
**M1 (0.75:0.50)^b^**	484.18 ± 6.04 *	31.77 ± 4.95 **
**M2 (0.75:0.12) ^b^**	496.21 ± 9.04 *	37.39 ± 9.39 **
**M3 (0.50:0.50) ^b^**	496.40 ± 10.79 *	36.18 ± 8.26 **
**M4 (0.50:0.12) ^b^**	511.88 ± 4.91	38.09 ± 7.68 **
**M5 (0.25:0.50) ^b^**	512.41 ± 5.39	45.83 ± 11.18 **
**M6 (0.25:0.12) ^b^**	514.30 ± 10.50	74.04 ± 16.82 **
**M7 (0.12:0.50) ^b^**	516.90 ± 6.02	85.515 ± 14.53 **
**M8 (0.12:0.12) ^b^**	520.18 ± 6.32	122.44 ± 24.81 *
**M9 (0.06:0.50) ^b^**	529.13 ± 4.90	130.81 ± 28.24 *
**M10 (0.06:0.12) ^b^**	534.72 ± 8.31	141.06 ± 19.66 *

^a^ Every value was detected by fluorescence assay in three independent treatments. Each concentration of the combined drugs was done in three times in each experiment, and the final fluorescence value is considered the mean and standard deviation (SD) of three independent experiments after subtraction of the background fluorescence for free parasitized RBCs and multiplied by 100.* *P* < 0.05 statistically significant differences between the combined-drug-treated group and the control group. **P < 0.05 statistically significant differences between the combined-drug-treated group and the both control group and PYR- treated group. PYR, pyronaridine tetraphosphate. ^b^ M1- 10 refers to the combinations of myrrh oil combined with PYR based on the calculated IC_50_ values obtained from the in vitro fluorescence assay.

**Table 5:** Growth inhibition effect of myrrh oil combined with luteolin on *B. divergens* and *B. bovis* parasites.

Group	Fluorescence Values (mean ± SD) ^a^	
	*B. divergens*	*B. bovis*
**Control**	447.33 ± 37.50	222.94 ± 39.10
**Luteolin IC_50_**	231.40 ± 16.02	119.35 ± 10.86
**M1 (0.75:0.50)^b^**	413.52 ± 23.38	2.57 ± 1.27 **
**M2 (0.75:0.12) ^b^**	420.86 ± 28.36	8.95 ± 1.91 **
**M3 (0.50:0.50) ^b^**	429.83 ± 27.96	22.76 ± 7.33 **
**M4 (0.50:0.12) ^b^**	425.425 ± 27.55	31.58 ± 8.19 **
**M5 (0.25:0.50) ^b^**	433.56 ± 29.78	100.89 ± 2.68 **
**M6 (0.25:0.12) ^b^**	435.37 ± 28.84	122.34 ± 28.41 *
**M7 (0.12:0.50) ^b^**	435.43 ± 31.26	140.94 ± 19.29 *
**M8 (0.12:0.12) ^b^**	434.18 ± 25.39	178.99 ± 8.20
**M9 (0.06:0.50) ^b^**	436.66 ± 27.88	185.54 ± 10.68
**M10 (0.06:0.12) ^b^**	448.03 ± 15.92	191.33 ± 18.37

^a^ Each value was detected by fluorescence assay in three independent experimental treatments. Each concentration of the combined drug was done in three times in each experiment, and the final fluorescence value is considered the mean and standard deviation (SD) of three independent experiments after subtraction of the background fluorescence for free-parasitized RBCs and multiplied by 100. * *P* < 0.05 statistically significant differences between the combined-drug-treated group and the control group. **P < 0.05 statistically significant differences between the combined-drug-treated group and the both control group and luteolin- treated group. ^b^ M1-10 refers to myrrh oil combined with luteolin based on the calculated IC_50_ values obtained from the fluorescence assay.
